# Psychometric evaluation of the Protection Motivation Theory scale in assessing fall protection motivation among older adults to reduce fall risk

**DOI:** 10.1186/s12877-023-04372-5

**Published:** 2023-10-31

**Authors:** Mei Fong Ong, Kim Lam Soh, Rosalia Saimon, Ing Khieng Tiong, Hasni Idayu Saidi, Manfred Mortell

**Affiliations:** 1 Department of Nursing, Universiti Putra Malaysia, Serdang, Selangor 43400 Malaysia; 2 Department of Nursing, Universiti Malaysia Sarawak, Kota Samarahan, Sarawak 94300 Malaysia; 3 Community Medicine and Public Health, Universiti Malaysia Sarawak, Kota Samarahan, Sarawak 94300 Malaysia; 4 Department of Geriatric Medicine, Institute of Sarawak Heart Centre/Sarawak General Hospital (Ministry of Health Malaysia), Kota Samarahan, Sarawak 94300 Malaysia; 5 Department of Biomedical Science, Universiti Putra Malaysia, Serdang, Selangor, 43400 Malaysia; 6 University of the Bahamas, Nassau, Bahamas

**Keywords:** Psychometric evaluation, Reliability and validity, Protection Motivation Theory scale, Older adults, Community older adults, Fall risk, Fall prevention, Behavioural change, Healthcare providers, Fall protection motivation

## Abstract

**Background:**

Protection Motivation Theory could be another potential and good framework that addresses essential elements in a behavioural change leading to positive fall protective behaviours. The positive behavioural change could reduce the risk of falls and improve the quality of life of the older community. The study aims to evaluate the reliability and validity of the culturally adapted Protection Motivation Theory scale for older adults' fall protection motivation or protective behaviours to reduce fall risk.

**Methods:**

A cross-sectional study was conducted to establish a psychometric instrument validation. A total of 389 participants aged 55 years and above were included. The study was conducted in Sarawak, Malaysia, from November 2021 to January 2022 in two phases, translation of the PMT Scale, cross-cultural adaptation, face validation and pre-testing of the PMT Scale. The participants were selected using multistage random sampling in a primary healthcare clinic. Data entry and statistical analysis were performed using IBM SPSS version 26 for exploratory factor analysis and SmartPLS version 3.3.7 for confirmatory factor analysis using partial least square structural equation modelling.

**Results:**

The Kaiser–Meyer–Olkin value was 0.760, Bartlett's sphericity test was significant and the total variance explained was 61%. It identified 31 items within eight dimensions of the Protection Motivation Theory scale. The Higher Order Constructs' measurement model indicates that the convergent and discriminant validity were established (Cronbach's alpha and composite reliability: ≥ 0.740; average variance extracted: 0.619 to 0.935 and Henseler's Heterotrait-Monotrait criterion for all constructs' discriminant validity: < 0.9). Test–retest for the intraclass correlation coefficient was 0.745. The model's coefficient of determination demonstrated R^2^ = 0.375.

**Conclusion:**

Overall, the Protection Motivation Theory Scale has established its reliability and validity for assisting older adults in the community. The Protection Motivation Theory Scale could be used in fall prevention interventions by promoting fall protective behaviours to reduce fall risk among community-dwelling older adults. The scale could assist healthcare providers in assessing the intention of older adults to use fall protective behaviours to reduce fall risk and serve as an alternative reference in developing fall prevention education in a fall prevention strategy.

**Supplementary Information:**

The online version contains supplementary material available at 10.1186/s12877-023-04372-5.

## Background

Falls remain a continuing health concern among the older population in most countries with an ever-increasing ageing population globally [1]. The consequences of falls among older individuals affect their well-being and social relationships, as well as implicate financial and care burdens on caregivers [1]. Furthermore, healthcare expenses dramatically increased for hospitalisation and treatment of older adults due to fall-related injuries [1].

A previous study's findings revealed a gap in fall management for older adults with a higher risk of falls in Malaysia due to a lack of design in fall prevention policies and inadequate training in fall management among healthcare professionals [2]. Therefore, the findings also indicated that most older individuals at risk or low risk of falls were most likely to be missed or excluded from fall prevention interventions. Furthermore, most healthcare providers or older individuals seldom communicate about falls or fall prevention due to the normalisation or stigmatisation of falls [2, 3]. Older adults refuse to talk about falls, which hinders their falls from family members or healthcare providers, as they perceive this event as part of their ageing process [4]. Therefore, these suggest that older adults need to be informed about falls and that healthcare providers are encouraged to communicate about falls. Furthermore, providing continuous education to older communities enhances their participation or uptake in fall prevention interventions [5].

Fall prevention interventions guided by theories promote positive behavioural change among older individuals [6]. The positive behavioural change could therefore reduce the risk of falls and improve the quality of life of the older community. However, many available interventions are still based on non-theoretical guided interventions [6]. Therefore, a well-designed fall prevention programme supported by a theoretical framework substantially improves older adults' knowledge and perception of fall threats while encouraging them to engage in preventive behaviour to reduce fall risk [6, 7]. Hence, the Protection Motivation Theory (PMT) could be another potential and good framework that conceptualises behavioural engagement [8, 9]. The PMT addresses essential elements such as a person's response towards threat appraisal, coping appraisal, fear and motivation in a behavioural change leading to positive fall protective behaviours [8, 9].

Fear, coping appraisal and motivation for fall prevention interventions are crucial factors contributing to older adults' engagement in fall preventive behaviour [10–13]. However, there has been no investigation of their self-efficacy, coping appraisal or protection motivation for fall prevention using the PMT scale or questionnaire in Malaysia, except on their knowledge and behaviour on fall prevention [14]. Hence, this PMT scale is beneficial in assessing the following components: coping appraisal in their fall prevention and the intention to engage in preventive behaviours.

PMT was developed by Rogers in 1975 and originally designed to test how fear influenced individuals to change their health behaviours or describe how individuals are motivated to react in a self-protective way towards a perceived health threat [8, 15]. The threat appraisal in PMT depends on the individual's belief in the seriousness of the problem, which is also known as perceived severity. Another component of the threat appraisal was perceived vulnerability. Older adults estimate their possibility of experiencing falls, such as they anticipate being at higher risk of falling if they do not adopt fall preventive behaviours. In perceived rewards (intrinsic/extrinsic), people believe in the positive aspects of their unhealthy behaviours. Thus, the motivation to participate in health-promoting behaviours is higher if the older adults have greater perceived severity and vulnerability with low perceived rewards [9].

Furthermore, response efficacy in coping appraisal occurs when an older adult assesses whether the protective behaviours are beneficial in overcoming the threat [8, 9]. In the self-efficacy component, individuals are confident that they can use their abilities to perform protective behaviour successfully. However, both efficacies are influenced by response cost. Older adults approximate and recognise the benefits of adopting fall preventive behaviours and considering the financial, effort, time and person are essential factors. Therefore, response efficacy and self-efficacy in coping appraisal are predicted to enhance the intention of protection behaviours, while response costs are expected to reduce the intention of protective behaviours.

**Fig. 1:**
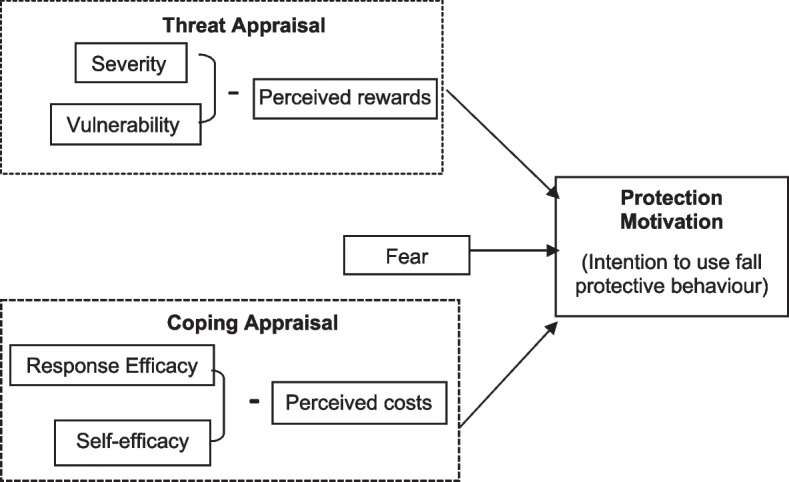
The PMT framework

Therefore, the PMT framework (Fig. 1) could be another option for healthcare providers to assess protective behaviours or motivation in applying preventive behaviours to reduce their fall risks. The original authors for the PMT scale had developed and tested among older Iranian adults [9]. However, no previous attempt was made to adapt the instrument measuring fall protection motivation among older adults in Malaysia.

## Methods

### Aims

The study aims to evaluate the reliability and validity of the culturally adapted Protection Motivation Theory scale for older adults' fall protection motivation or protective behaviours to reduce fall risk.

### Study design

A cross-sectional study was conducted in Sarawak, Malaysia, from November 2021 to January 2022. The study consisted of two phases, (i) translation of the PMT Scale, cross-cultural adaptation and face validation, (ii) pre-testing of the PMT Scale.

### Sample

Three hundred eighty-nine older adults aged 55 years and above were identified and included from a local primary healthcare clinic in Sarawak. Multistage random sampling was adopted to identify and select a primary healthcare clinic and participants from the clinic. This sampling was appropriate to be adopted for large or dispersed populations [16]. There was an estimation of 12 divisions governed by the Sarawak state's administration. A primary healthcare clinic was randomly selected from a division after a division was randomly identified from the 12 divisions registered within the Sarawak State Health Department. Next, 10 to 30 participants registered with the clinic were randomly chosen in each community within 18 settlements receiving healthcare services from this primary healthcare clinic.

The sample size was determined based on the minimum ratio between items per response: one item to five responses for exploratory factor analysis (EFA) [17]. Several authors justified that a ratio of one item to five responses was able to approximate about 40% of samples with correct structures [18]. They also recommended a larger sample of 300 or applying a higher ratio of one item to ten or twenty responses for better sample size estimation [18]. On the contrary, other authors suggested that samples in the range of 100–200 were appropriate with well-determined factors, such as the main factors defined by many indicators or indicator variables with loadings > 0.80 and communalities within the range of 0.5 or above [19]. They also stated that if the communalities fall into the range of 0.40 to 0.70, then the sample size should be at least 200 [19]. The instrument tool used in this study was adapted from a previous study with constructs or factors derived from an established theory. Therefore, it was considered to have well-determined factors. This study's communalities of factor analysis ranged between 0.5 and 0.84, with each factor comprising several indicators. Hence, 184 participants were considered as an acceptable sample size to be included for EFA, in which a total of 389 participants were randomly split into the first half for EFA and the second half, with a total of 195 participants were included in the confirmatory factor analysis (CFA) using the partial least square structural equation modelling (PLS-SEM) [17]. In addition, the total number of participant has met the minimum sample size of 160 for PLS-SEM analysis [20].

The inclusion criteria included community-dwelling older adults at the primary care clinic of Kota Samarahan aged 55 years old and above who could read, write or understand Malay or English. Those suffering from mental health problems were excluded from the study.

### Validity and reliability of the instrument

Validity was assessed on an instrument tool that correctly measures what it intends to measure [21]. This assessment includes content and construct validity [22]. Both validities refer to the degree to which instrument content sufficiently reflects the construct being measured and to which a set of variables represents the construct to be measured [17]. Therefore, content validity was assessed using the panel committees' expert opinions on the items within the instrument and rated according to their equivalent in the content validity index (CVI). Next, the construct validity was inquired using the EFA and CFA.

Reliability was tested to ensure that the instrument tool has the ability to reproduce a consistent result or refer to stability, internal consistency and equivalence of a measure [22]. Several examples of the tests were used, such as the intraclass correlation coefficient (ICC), mainly used to assess continuous variables stability while also considering the measurement errors [23]. Secondly, another assessment was test–retest reliability, which was performed to estimate the consistency of measurement repetition [22]. Thirdly, Cronbach's alpha coefficient was a commonly used assessment to determine the instrument tool's internal consistency [17].

The questionnaire consists of two parts and was approved for use by the original authors [9, 24]. Part I is the adapted and modified version of the participants' sociodemographics: age, gender, educational level, ethnicity and fall history. Meanwhile, the adapted Part II questionnaire consists of 35 items with eight constructs of the PMT scale. The PMT scale was scored using a 5-point Likert Scale, from strongly disagree to strongly agree, ranging from one to five for perceived sensitivity/severity, self-efficacy, response efficacy and perceived rewards. Meanwhile, another five-point Likert Scale ranging from not at all (1), a little (2), somehow (3), much (4), to too much (5) rated for fear, perceived costs and protection motivation.

#### Phase I: Translation of the PMT scale, cross-cultural adaptation and face validation

**Fig. 2:**
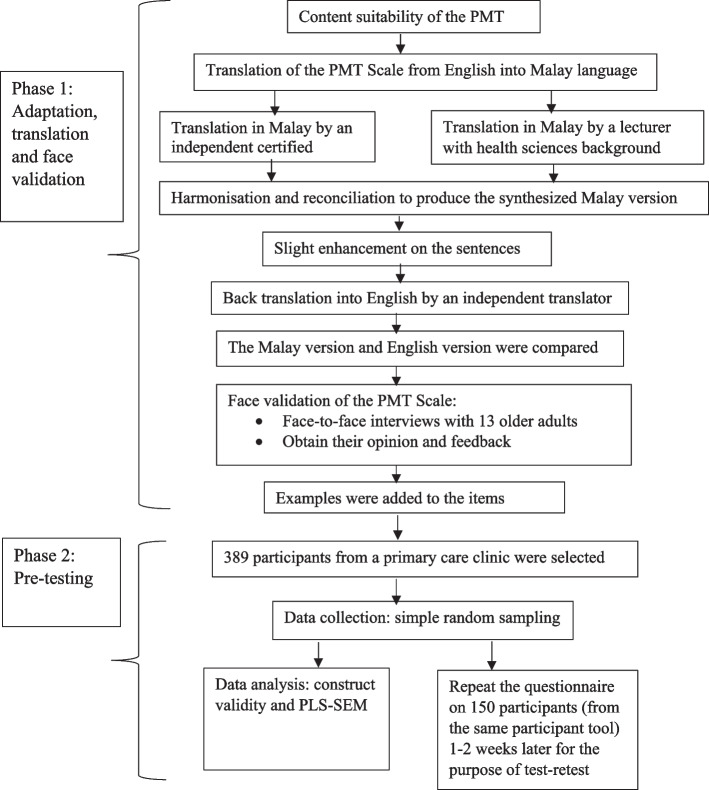
Flow chart of the adaptation, translation, face validation and pre-testing process of the PMT Scale

The translation and adaptation process were referred to guidelines of WHO [25], Gjersing, Caplehorn, and Clausen [26] and Beaton, Bombardier [27] (as illustrated in Fig. 2). First, five professionals consisting of three lecturers in health sciences, a physician and a geriatrician reviewed the original PMT scale for content suitability according to the local setting. Next, three panel committees assessed and rated the instrument's CVI.

Second, the questionnaire was translated into the Malay language by an independent certified translator who is bilingual (Malay or English). In addition, another translator with a health sciences background has independently translated the scale into Malay. The study members then reviewed and clarified both versions of the translated Malay questionnaire. Fall is defined during the harmonisation process based on the WHO [1] and WHO [28] for the Malay translation. The contents were identical to the original version and apart from that, a slight sentence enhancement was made. Another independent translator then translated it back into English. In the final phase of this process, the committee members reviewed and assessed the original instrument by comparing the translated and back-translated questionnaires for accuracy.

Thirteen older adults aged 55 years old and above from various educational backgrounds and ethnicities were randomly selected for face validation to validate the cultural appropriateness [25]. Participants were interviewed individually based on the PMT scale. The principal investigator requested the participants to advise of any unclear, confusing statements or scoring methods in the questionnaire [25], such as their thoughts about a particular question that was being asked, what were their thoughts or understanding of a question, phrase or term used when it was read out to them or alternative words to conform among them [25]. A majority of them stated they could follow and understand the questions. However, they suggested including examples of older adults' daily routines or activities in several statements, especially for response efficacy, self-efficacy, perceived costs and protection motivation items, to enhance their understanding of the questions. Older adults were also asked for the rationales of their selected answers. Their responses were compared between the first and second responses. Their second response was collected within 14 days after the first administration to ensure consistency between both responses [25]. Several subscales and questions were later improved by adding examples, including seven items from protection motivation, self-efficacy, response efficacy, perceived costs, and perceived severity, which were enhanced with sentence adjustment or additional examples.

#### Phase II: Pre-testing of the PMT Scale

Older adults aged 55 years and above have participated in this cross-sectional survey. The final version of the PMT Scale was tested among 389 participants who had fulfilled the same inclusion criteria. Furthermore, about one hundred fifty participants from the same population were included for a test–retest of the PMT scale. They were requested to answer a similar questionnaire for the second time, ranging from seven to 14 days later [22].

### Data collection

Data collection was carried out when the participants were at the clinic, followed by a test–retest assessment performed at their homes. The participants were informed about the purpose of the study and their consent was obtained after fulfilling the inclusion or exclusion criteria. It was conducted through (i) face-to-face interviews and (ii) a self-administered questionnaire for those willing to answer independently. Both interviews and self-administered questionnaires used a similar questionnaire. They were also informed and allowed to withdraw from participating in the study without any penalties.

The principal investigator had briefed assistant investigators on the content of the questionnaires before the data collection started and investigators calibration was also conducted two weeks later to ensure the consistency of the data collected between the assistant investigators. Next, participants were invited to participate in the study and offered to self-administer the questionnaire. Most participants requested or preferred to be interviewed and some were being assisted during the self-administered questionnaires. The assistant investigators selected and interviewed participants whilst monitored by the principal investigator. Participants were also informed that there would be a second visit from the investigators at their home. Therefore, their contact numbers were obtained to schedule a second test–retest assessment visit. The investigators also checked for any incomplete information at the end of the interviews or during the collection of the questionnaire. The interview session lasted about 30 to 35 min.

### Ethical considerations

The ethical approval to conduct the study was obtained from the National Medical Research Register, Medical Research and Ethics Committee, Ministry of Health Malaysia (NMRR-21–1680-61095) and the Sarawak State Health Department. All participants were provided with oral and written informed consent. Their identity was kept confidential and not entered into the database.

### Data analysis

Data entry and statistical analysis were performed using IBM SPSS version 26 for EFA and SmartPLS version 3.3.7 for PLS-SEM. The Mahalanobis's Distance was also performed to identify extreme outliers and ten participants were deleted from the analysis with the Mahalanobis multivariate outlier test result with significant observations (*p* < 0.001) [29]. EFA was tested for the adapted version of the instrument tool from another language to the local language and aimed to explore a new measure in determining the factors within an unfactorised measure [17, 30]. Meanwhile, CFA using PLS-SEM was tested to confirm a pre-existing factor structure that has already been determined [17, 30].

PLS-SEM was considered an appropriate analytical method with models that consisted of many constructs and indicators, in which the study has eight constructs with thirty-five indicators in total [31]. Additionally, this approach was performed to predict and describe the essential target constructs, identify the important driver constructs, then allow one to form a higher-order construct to explain a relationship between a newly formed indicators and constructs [31]. Furthermore, PLS-SEM was considered as soft-modeling due to its high flexibility in adjusting assumptions of data distributions [32]. Hence, Smart PLS is considered as an appropriate software for analysing structural equation modelling (SEM) [33].

#### Exploratory factor analysis

EFA using principal component analysis (PCA) with oblique rotation (Promax) and Eigenvalues of > 1 was performed to identify the constructs and their dimensions. The value for significance was only limited to 0.40 and above to be accepted. The item loading selection was determined when the i) primary loading was > 0.40, ii) cross-loadings were > 0.2 between the primary and secondary loading, iii) the minimum of two items was necessary to be loaded in a factor, iv) the relevance of items in a factor loading [17].

#### Confirmatory factor analysis: PLS-SEM

The analysis aims to examine the convergent validity using the average variance extracted (AVE: > 0.50) and composite reliability (CR: > 0.70) [32, 34]. The discriminant validity was analysed using Henseler's Heterotrait-Monotrait (HTMT: < 0.90) correlation ratio [32, 34]. Higher-order constructs (HOCs) were also performed to merge the self-efficacy and response efficacy for the coping appraisal construct, vulnerability and severity for the threat appraisal construct. The blindfolding assessment was performed to identify the protection motivation's predictive relevance (Q^2^: > 0), followed by PLSpredict to assess the model's out-of-sample predictive power [35].

## Result

### Demographic profile

A total of 379 participants were included in the study. The mean age was 65.42 (8.547). Female consisted of more than half of the participants (54.6%) and male (45.4%), most of them had low literacy and had attained primary education (45.1%), this was followed by participants who had not received formal education (24.3%), had achieved secondary education (21.9%) and had completed higher education (8.7%). In terms of ethnicity, there were more Malays (71.0%) than Iban (20.6%), Chinese (4.7%) and other races (3.7%). About 44.8% of them experienced falls, including those who had started falling more than one year ago (23.2%) and falls in the previous year (21.6%).

### Test–retest reliability testing and content validity index

The interclass correlation (ICC) among 150 older participants shows a value of 0.745, indicating satisfactory test–retest reliability and each construct demonstrated ICC ranging from 0.590 to 0.788 [36]. Meanwhile, the CVI for the instrument was 0.95, indicating a high index [22].

### Exploratory factor analysis

**Fig. 3:**
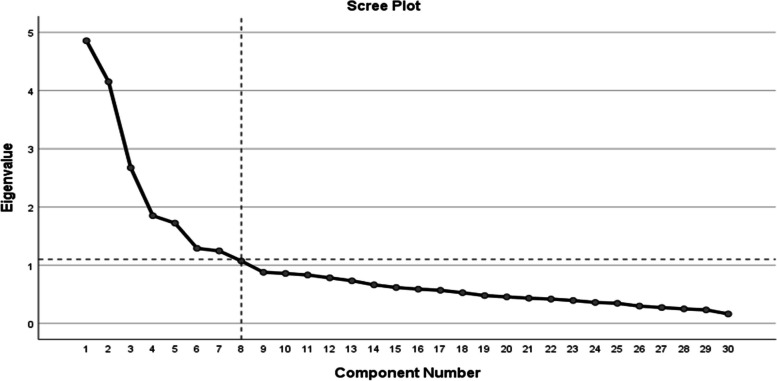
Scree plot of exploratory factor analysis for the PMT Scale

The KMO value for EFA shows 0.760 and Bartlett's test of sphericity with a significant *p*-value < 0.001 with a total variance explained was 61% (Table 1). In addition, all items in each factor loading showed values ranging from 0.744 to 0.767. In the PMT scale, eight constructs consisted of vulnerability, severity, response efficacy, self-efficacy, perceived rewards, perceived costs, fear and protection motivation (Fig. 3). Four items were excluded (two items from perceived costs, each item in vulnerability and self-efficacy, respectively) since their factor loading was less than 0.4 or items fall between two constructs with cross-loading of a ratio was less than 0.2. The self-efficacy constructs (SE5: asking the doctor about the side effect of the medication) was loaded within the perceived reward. Furthermore, the cross-loading ratio of the item was less than 0.2 between the two factors. The other two items have factor loading < 0.4. Although PC3 had a factor loading of 0.435, its Cronbach's Alpha was 0.231, which was lower than the acceptable range. The protection motivation, fear and response efficacy were individually loaded into their constructs. Finally, 31 items within eight factors were considered as compared to 35 items from the original version.

**Table 1:** Assessment of construct validity using EFA

	Factor 1	Factor 2	Factor 3	Factor 4	Factor 5	Factor 6	Factor 7	Factor 8
PM1	0.875							
PM3	0.795							
PM4	0.772							
PM5	0.758							
PM2	0.743							
RE2		0.742						
RE1		0.739						
RE3		0.636						
RE4		0.548						
RE5		0.473						
RE6		0.455						
F2			0.911					
F3			0.864					
F1			0.832					
S6				0.896				
S4				0.660				
S5				0.514				
S3				0.402				
V1					0.892			
V3					0.864			
S1					0.576			
S2					0.532			
V2					***0.314***			
PR2						0.753		
PR1						0.613		
SE5					***-0.307***	***0.571***		
PC4							0.857	
PC5							0.826	
PC1							0.859	
PC3							***0.435***	
PC2							***0.310***	
SE4								0.851
SE2								0.915
SE3								0.557
SE1								0.493

The Italic and bold numbers denote the deletion of items due to low values than the threshold range of factor loading/α/AVE

### Confirmatory factor analysis

The Cronbach's alpha for the measurement model of low-order constructs ranged from 0.706 to 0.931 (> 0.70), CR ranged from 0.821 to 0.967 (> 0.70), AVE ranged from 0.538 to 0.935 (> 0.50), indicating that the convergent validity of the model was appropriate (Table 2a) [32, 34]. The HTMT inference criterion and the discriminant validity were also established.

**Table 2:** Assessment of Measurement Model for (a) Low Order Construct and (b) Higher Order Construct

(a)Low Order Construct						(b) Higher Order Construct				
**Latent variable**	**Item**	**Outer loading**	**Cronbach's Alpha**	**Composite reliability**	**AVE**	**Latent variable/ Item**	**Outer loading**	**Cronbach's Alpha**	**Composite reliability**	**AVE**
Fear	F1	0.916	0.913	0.945	0.852	F1	0.916	0.913	0.945	0.852
	F2	0.937				F2	0.937			
	F3	0.917				F3	0.916			
PM	PM1	0.849	0.845	0.890	0.619	PM1	0.849	0.845	0.890	0.619
	PM2	0.735				PM2	0.821			
	PM3	0.799				PM3	0.799			
	PM4	0.844				PM4	0.844			
	PM5	0.696				PM5	0.697			
Perceived costs	PC1	0.772	0.779	0.872	0.695	PC1	0.744	0.779	0.872	0.695
	PC4	0.823				PC4	0.821			
	PC5	0.902				PC5	0.902			
Perceived rewards	PR1	0.966	0.931	0.967	0.935	PR1	0.966	0.931	0.967	0.935
	PR2	0.969				PR2	0.968			
Response efficacy	RE1	0.850	0.884	0.912	0.635	Response efficacy	0.949	Coping appraisal = 0.888	Coping appraisal = 0.947	Coping appraisal = 0.899
	RE2	0.845								
	RE3	0.865								
	RE4	0.812								
	RE5	0.763								
	RE6	0.620								
Self efficacy	SE1	0.772	0.822	0.882	0.651	Self efficacy	0.947			
	SE2	0.818								
	SE3	0.796								
	SE4	0.839								
Severity	S3	0.794	0.793	0.864	0.613	Severity	0.953	Threat appraisal = 0.740	Threat appraisal = 0.874	Threat appraisal = 0.778
	S4	0.765								
	S5	0.772								
	S6	0.800								
Vulnerability	V1	0.580	0.706	0.821	0.538	Vulnerability	0.805			
	V3	0.719								
	S1	0.833								
	S2	0.777								

The Higher Order Constructs' (HOCs) measurement model indicates that the Cronbach's alpha and CR were 0.740 and above, AVE ranged from 0.619 to 0.935 and HTMT criterion for all constructs' discriminant validity was less than 0.9 (Table 2b). Thus, the convergent and discriminant validity were established [37]. In addition, the full collinearity formed a variance inflation factor (VIF) below 3.30, suggesting that the common method bias is within the normal range [38].

**Fig. 4:**
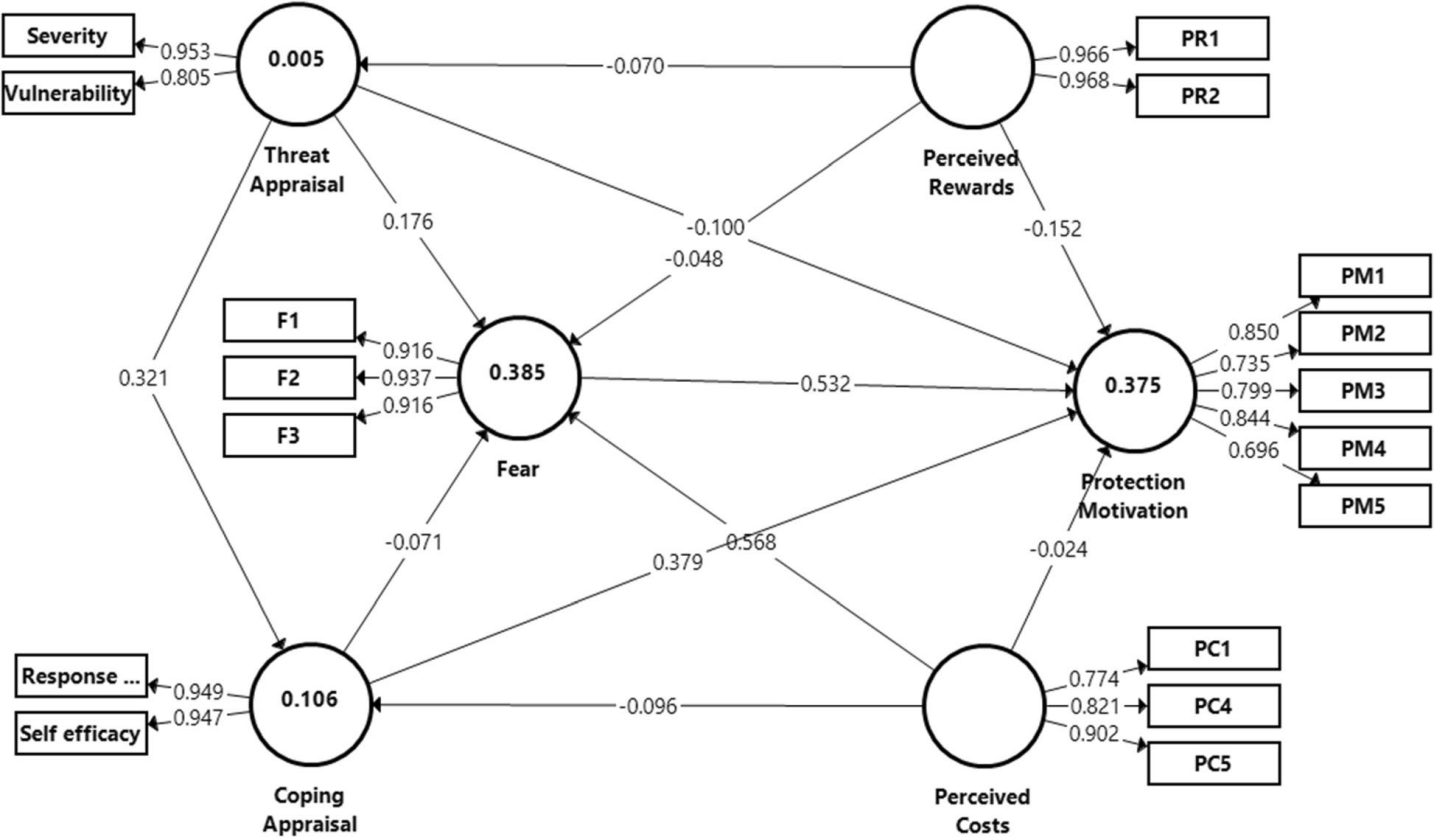
The PLS-SEM of the PMT Scale

Figure 4 displays the model's coefficient of determination (R^2^ = 0.375) and indicates almost moderate in-sample predictive power [31]. The path coefficient between a coping appraisal (β = 0.379, t = 5.264, *p* < 0.001, effect size (ƒ^2^) = 0.198), fear (β = 0.532, t = 7.480, *p* < 0.001, ƒ^2^ = 0.279), perceived rewards (β = -0.152, t = 2.548, *p* = 0.001, ƒ^2^ = 0.035) and protection motivation is statistically significance (Table 3, Fig. 4). A statistical significant path coefficient is also observed between perceived costs (β = 0.568, t = 10.540, *p* < 0.001, ƒ^2^ = 0.512), threat appraisal (β = 0.176, t = 2.954, *p* = 0.003, ƒ^2^ = 0.044) and fear. The threat and coping appraisals (β = 0.321, t = 4.323, *p* < 0.001, ƒ^2^ = 0.114) presented statistically insignificance correlation. On the contrary, perceived rewards and threat appraisal (β = -0.070, t = 0.748, *p* = 0.454), perceived costs and coping appraisal (β = -0.096, t = 1.295, *p* = 0.196) were statistically non-significance negative correlation. Hair et al. [31] stated that 0.02, 0.15 and 0.35 denote small, medium and large effect sizes, respectively.

**Table 3:**
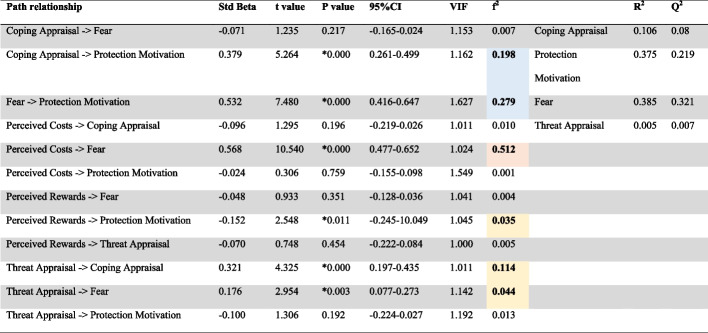
Assessment of structural assessment

* Value denotes statistical significant path coefficients, *p* < 0.05

VIF < 3.30, f^2^: 0.02-0.15 (small), 0.15-0.35 (medium), 0.35 > (large)

Large effect size;  Medium effect size;  Small effect size

The predictive relevance (Q^2^) for the protection motivation was 0.219, suggesting that the model has predictive power relevance [31]. Most of the item values of protection motivation, threat appraisal, coping appraisal and fear in the PLS-SEM model have a lower prediction error (i.e., RMSE) than the linear model (LM). Therefore, the model demonstrates medium predictive power after considering the PLS-SEM versus linear regression model [35]. The global fit measure (GoF) was 0.416, indicating better power than the baseline values (GoFsmall = 0.1, GoFmedium = 0.25, GoFlarge = 0.36) [39].

There was also a mediation effect between threat appraisal and protection motivation via coping appraisal (β = 0.129, t = 3.034, *p* = 0.002), fear between perceived costs and protection motivation (β = 0.302, t = 5.924, *p* < 0.001) and fear between threat appraisal and protection motivation (β = 0.094, t = 2.689, *p* = 0.007). All these constructs show a positive, statistically significant direct and indirect relationship, indicating a complementary partial effect.

## Discussion

The results conclude that the PMT Scale is a reliable and valid instrument by excluding four items from the original version. The four items were excluded after EFA and PLS-SEM demonstrated weak factor loading and low values for Cronbach's Alpha (< 0.4), CR (< 0.4) and AVE (< 0.5). The deleted four items comprised one item from perceived vulnerability, self-efficacy (1 item) and perceived costs (2 items). The deleted items were (i) fall is not common and it may not happen to me, (ii) I can ask my doctor about the side effects of my medication, (iii) fall prevention facilities and equipment are expensive and (iv) I do not know how to prevent falls. The other two items of the perceived severity subscale (i.e., longer hospitalisation after falls and sustained fractures/injuries after falls) were grouped into the vulnerability construct. Their perceived vulnerability towards both items could be due to their advanced age and frailty state [3].

The omission of two items in perceived costs was probably due to a lack of awareness towards fall prevention interventions or low health literacy of individuals [40]. The majority of older adults were unsure or did not perceive either the high price of equipment or not knowing how to prevent falls as factors that might determine their risk of falls. This is probably caused by their inadequate knowledge of fall prevention interventions or not knowing the benefits of fall prevention equipment or assistive devices to reduce fall risks [41]. In addition, this also highlighted the influence of their financial constraints and perception on priorities to improve their living conditions for home modifications [42]. It could also be a result of their denial of accepting any forms of fall-related advice, leading them to be ignorant [2].

A probability relationship between their low education attainment and the omission of an item within the vulnerability construct might be due to the majority of older adults never experienced falls and thus, reported difficulty in determining the seriousness of falls. In some circumstances, they probably perceive falls as a non-medical issue or as severe as requiring a doctor's attention [43]. Thus, older adults should be educated on fall awareness, including fall threats and consequences.

In addition, their lack of awareness of fall risk factors also influenced whether to ask or not to ask about their given medications. The time constraints during the consultation and obtaining care from several physicians added barriers to ask about the side effects of the drugs [44]. Older adults also might not receive adequate information on managing their medications due to a lack of clinical skills or unfamiliarity with managing falls by healthcare providers [2]. Furthermore, the lack of healthcare system support in providing training for healthcare providers on falls and their preventions contributed to their low confidence in managing or identifying falls among older adults [32].

Overall, this study's R^2^ (0.375) value was higher than the original study conducted by Taheri-Kharameh and colleagues [9], R^2^ = 0.265. The higher R^2^ value in this study could be due to the focus of the study on older adults' intention to use protection motivation or protective behaviours. Furthermore, the greater their coping mechanism (self-efficacy and response efficacy) of falls, the higher their protection motivation. Both coping appraisal and fear play important roles in determining the older adults' using fall protective behaviours. Thus, it is essential for healthcare providers and caregivers to assess, facilitate and support older adults' coping strategies in fall prevention. In addition, the community health promotion efforts by nurses should also emphasise on promoting older individuals' self-efficacy, such as integrating to reinforce their intent, which eventually lead to their engagement [15].

Fear was more strongly associated with older adults' protection motivation than in the original study. This finding suggests that fear stimulates protective factors among these older individuals since it has been conceptualised as an affective state defending one against danger and motivating people to practise protective behaviours [8, 15]. Conversely, their greater fear could be due to fear of falling or losing their independence once they fall [12]. In addition, it could also be due to their low self-efficacy, little ability to prevent falls or lack of knowledge in dealing with fall prevention, causing an increase in their fear [12, 45, 46]. Therefore, providing continuous fall prevention education and support in their coping appraisal by healthcare providers and family members may reduce their fear.

On the contrary, threat appraisal has a similar outcome as reported in the original research, in which protection motivation was less expected to be influenced by emotional factors. This result explained that they know or are aware that they are at risk of falls but not motivated to do any preventive behaviours [9]. According to Rippetoe and Rogers [47], the increase in perceived threats may have various outcomes. In some circumstances, it promotes their intent to engage in preventive behaviour. However, in other situations, it resulted in denial and avoidance [35]. Hence, it was crucial to educate them about fall risks. The finding of this study also confirmed that threat appraisal was associated with their coping appraisal. The more they are aware of the fall threats, the more it enhances their coping strategies.

Furthermore, this study produces a similar finding for perceived rewards and costs to the initial research, where an increase in rewards or costs resulted in low threat or coping appraisal. The result has concurred with the concept of the PMT that older people would cope or react to threats better if they perceived fewer costs or rewards [8, 9]. However, the greater they are perceived in costs, the better it enhances their fear and leads to higher protection motivation.

The ƒ^2^ value of the protection motivation in this study was almost similar to the original finding. The ƒ^2^ will be increased if the exogenous construct significantly contributes to explaining an endogenous construct [31]. Conversely, the Q^2^ value of more than zero summarises the predictive accuracy of the endogenous construct as indicated in the protection motivation construct [17]. In other words, it signifies that the model has predictive power for future cases or observations and it was supported in the PLSpredict assessment that resulted in a medium predicting relevance.

Therefore, the PMT Scale could provide an alternative reference for healthcare providers in selecting evidence-based fall prevention strategies in clinical practice. The PMT framework underpinned within this scale would be beneficial guidance for healthcare providers when integrating its components into the fall prevention education module in the primary care setting, particularly in highlighting older individuals' threat appraisal, fear, perceived costs and coping appraisal elements. The outcome of this study enables healthcare providers to integrate the fear, coping appraisal and protection motivation to promote their independence by educating them to recognise their fall risks and how to prevent those modifiable risks. Assessing their protection motivation also allows healthcare providers to determine their strengths and weaknesses for intention to engage in preventive behaviour and appropriate coping strategies can be recommended.

However, future recommendations are to further validate this PMT scale to other older populations without any restrictions on a history of mental illnesses. In addition, future psychometric properties testing of this PMT scale is recommended to be tested and compared to the findings among older people in urban settlements as more than half of the older participants in this study have not received a formal education or only completed elementary education. Further study is suggested to assess older adults' protection motivation and their outcomes in participating in preventive behaviours. However, this scale is time-consuming if it were to be practised in the clinical setting, thus, the scale is more suitable to be used among older adults potentially at risk for falls for further fall prevention management. In addition, the current version of the PMT scale is to be modified into a shorter version based on the related components of this PMT scale to suit the clinical practicality and needs.

### Limitations

Several limitations were also noted in this study. First, a cross-sectional study may limit participants' information on previous falls due to recall bias. Second, it is important to note that this study focuses only on community-dwelling older adults' intention to use fall-protective behaviours. Thus, it may not be able to assess their performance on actual fall preventive behaviours. Furthermore, the findings could not be generalised to the entire older population due to the exclusion of older participants with mental illnesses. The deletion of four items in the final scale was also probably due to the majority of the older adults in the populations coming from lower education backgrounds and associated with low health literacy, which affected their answer choices.

## Conclusion

Generally, the PMT Scale is reliable and valid for community-dwelling older people. The EFA and PLS-SEM results indicated that the PMT Scale has better convergent and discriminant validity by omitting four out of 35 items in this study. The Protection Motivation Theory Scale could serve as an alternative reference for healthcare providers in developing fall prevention education in the primary care setting and integrating it into the routine practice as the screening tools in primary care settings for assessing older adults' intention to use fall protective behaviours to reduce fall risk. Healthcare providers could also emphasise on promoting the older individuals' self-efficacy or coping methods, fear and independence living in fall prevention programmes to enhance their fall protective behaviours.

### Supplementary Information

**Additional file 1.**

## Data Availability

The datasets used and/or analysed during the current study are available from the corresponding author on reasonable request.
