# Supplementary material for: Psychometric evaluation of the Protection Motivation Theory scale in assessing fall protection motivation among older adults to reduce fall risk
Source: BMC Geriatr. 2023 Oct 31;23:703. doi: 10.1186/s12877-023-04372-5 (PMC10617071; doi:10.1186/s12877-023-04372-5)
Supplement: Supplementary file 1 — Additional file 1. [file 12877_2023_4372_MOESM1_ESM.docx]

Bahagian II: Persepsi Terhadap Terjatuh

Sila bulatkan setiap pernyataan yang anda rasa wajar.

**Contohnya:**

| Sangat setuju | Setuju | Kurang pasti | Tidak setuju | Sangat tidak setuju |
| --- | --- | --- | --- | --- |
| **5** | **4** | **3** | 2 | **1** |
| Tidak sama sekali | Jarang-jarang | Sedikit sebanyak | Kerap | Terlalu kerap |
| 1 | 2 | 3 | 4 | 5 |

Sila baca pernyataan-pernyataan berikut dan sejauh mana anda setuju atau tidak setuju dengan petikan dibawah.

| Bil. | Item  Soalan no. 1-2 | Sangat setuju | Setuju | Kurang pasti | Tidak setuju | Sangat tidak setuju |
| --- | --- | --- | --- | --- | --- | --- |
|  | Saya mungkin terjatuh juga di masa depan. | 5 | 4 | 3 | 2 | 1 |
|  | Kemungkinan untuk saya jatuh adalah lebih tinggi berbanding kawan-kawan saya. | 5 | 4 | 3 | 2 | 1 |
| Soalan no. 3-8  *Question no. 3-8* | | Sangat setuju | Setuju | Kurang pasti | Tidak setuju | Sangat tidak setuju |
| 3. | Sekiranya terjatuh, saya perlu tinggal di hospital lebih lama. | 5 | 4 | 3 | 2 | 1 |
| 4. | Sekiranya terjatuh, saya akan mengalami keretakan tulang dan kecederaan. | 5 | 4 | 3 | 2 | 1 |
| 5. | Kejadian terjatuh akan membuatkan saya murung, takut, dan cemas. | 5 | 4 | 3 | 2 | 1 |
| 6. | Kejadian terjatuh membuatkan saya perlu bergantung kepada orang lain dalam melakukan urusan seharian. | 5 | 4 | 3 | 2 | 1 |
| 7. | Sekiranya terjatuh, saya akan mengalami sakit teruk. | 5 | 4 | 3 | 2 | 1 |
| 8. | Kejadian terjatuh sangat membebankan keluarga (contohnya, kos rawatan/kemasukan ke hospital, beban untuk menjaga saya). | 5 | 4 | 3 | 2 | 1 |
| Soalan no. 9-11  *Question no. 9-11* | | Tidak sama sekali | Jarang-jarang | Sedikit sebanyak | Kerap | Terlalu kerap |
| 9. | Apabila terfikir tentang terjatuh, saya berasa cemas. | 1 | 2 | 3 | 4 | 5 |
| 10. | Saya berasa takut apabila berfikir tentang terjatuh. | 1 | 2 | 3 | 4 | 5 |
| 11. | Apabila terfikir tentang terjatuh, saya berasa sedih. | 1 | 2 | 3 | 4 | 5 |
| Soalan no. 12-15  *Question no. 12-15* | | Sangat setuju | Setuju | Kurang pasti | Tidak setuju | Sangat tidak setuju |
| 12. | Walaupun tergesa-gesa, saya boleh mengerjakan sesuatu dengan berhati-hati. | 5 | 4 | 3 | 2 | 1 |
| 13. | Saya boleh menggunakan tongkat/alat bantu berjalan atau alat sokongan lain, sekiranya perlu, walaupun saya kelihatan seperti tidak berupaya menggunakannya. | 5 | 4 | 3 | 2 | 1 |
| 14. | Saya dapat mencegah daripada terjatuh dengan mengamalkan perilaku pencegahan,  contohnya: menggunakan tongkat, pakai kasut/sandal/selipar yang sesuaim dan elak menggunakan kerusi/perabot untuk memanjat ketika mencapai barang. | 5 | 4 | 3 | 2 | 1 |
| 15. | Saya boleh mengelak daripada keluar sewaktu berlakunya ribut, hujan atau pada hari yang sibuk/ tempat sesak sekalipun perlu. | 5 | 4 | 3 | 2 | 1 |
| Soalan no. 16-18  *Question no. 16-18* | | Tidak sama sekali | Jarang-jarang | Sedikit sebanyak | Kerap | Terlalu kerap |
| 16. | Tiada alatan tersedia yang boleh mencegah saya daripada terjatuh (contoh: tongkat bantu berjalan, kerusi roda dan lain-lain). | 1 | 2 | 3 | 4 | 5 |
| 17. | Saya malu menggunakan peralatan sokongan seperti tongkat. | 1 | 2 | 3 | 4 | 5 |
| 18. | Saya tidak selesa meminta tolong daripada orang lain sekiranya perlu (contohnya, memerlukan bantuan untuk bergerak/menggerakkan badan, menukar lampu mentol dan mendapatkan sesuatu pada ketinggian yang sukar dicapai). | 1 | 2 | 3 | 4 | 5 |
| Soalan no. 19-24  *Question no. 19-24* | | Sangat setuju | Setuju | Kurang pasti | Tidak setuju | Sangat tidak setuju |
| 19. | Membuat perubahan di kediaman seperti menambahbaik pencahayaan (contoh: menukar/menambah mentol atau lampu) boleh mengelakkan seseorang daripada terjatuh. | 5 | 4 | 3 | 2 | 1 |
| 20. | Kejadian terjatuh boleh dicegah dengan menggunakan tongkat atau alat bantuan apabila perlu. | 5 | 4 | 3 | 2 | 1 |
| 21. | Dengan mengambilkira bahaya dalam menyiapkan sesuatu, kejadian terjatuh boleh dicegah. | 5 | 4 | 3 | 2 | 1 |
| 22. | Kejadian terjatuh boleh dicegah dengan mengelakkan diri daripada keluar sewaktu berlakunya ribut, hujan, atau pada hari yang sibuk. | 5 | 4 | 3 | 2 | 1 |
| 23. | Bertanyakan kepada doktor tentang kesan sampingan ubat yang diambil dapat mencegah saya daripada terjatuh. | 5 | 4 | 3 | 2 | 1 |
| 24. | Saya boleh mencegah daripada terjatuh dengan berhati-hati semasa urusan seharian saya. | 5 | 4 | 3 | 2 | 1 |
| Soalan no. 25-26  *Question no. 25-26* | | Sangat setuju | Setuju | Kurang pasti | Tidak setuju | Sangat tidak setuju |
| 25. | Saya ingin berdikari dalam melakukan sesuatu. | 5 | 4 | 3 | 2 | 1 |
| 26. | Saya berasa seronok apabila dapat menyiapkan sesuatu dengan pantas. | 5 | 4 | 3 | 2 | 1 |
| Soalan no. 27-31  *Question no. 27-31* | | Tidak sama sekali | Jarang-jarang | Sedikit sebanyak | Kerap | Terlalu kerap |
| 27. | Kini, saya merancang untuk lebih berhati-hati dalam melakukan sesuatu. | 1 | 2 | 3 | 4 | 5 |
| 28. | Saya merancang untuk membuat beberapa perubahan di kediaman untuk mengelak daripada terjatuh (contoh: susun semula barang di rumah, elakkan halangan di sepanjang laluan, guna tikar yang tidak menyebabkan tergelincir dll). | 1 | 2 | 3 | 4 | 5 |
| 29. | Saya merancang untuk tidak keluar sewaktu berlakunya ribut, hujan, atau pada hari yang sibuk. | 1 | 2 | 3 | 4 | 5 |
| 30. | Saya memutuskan untuk bertanya doktor tentang kesan sampingan ubat yang diberi atau diambil untuk mengelak daripada terjatuh. | 1 | 2 | 3 | 4 | 5 |
| 31. | Saya merancang untuk menggunakan tongkat atau alat bantuan sekiranya perlu. | 1 | 2 | 3 | 4 | 5 |
